# Meniscal tear of the knee causing cystic adventitial disease of popliteal artery: support for the synovial theory

**DOI:** 10.1093/bjrcr/uaaf015

**Published:** 2025-03-17

**Authors:** Ruhaid Khurram, Rashed Al-Khudairi, Parag Jaiswal, Helen Marmery

**Affiliations:** Department of Radiology, Royal Free Hospital, London NW3 2QG, United Kingdom; Department of Radiology, Royal Free Hospital, London NW3 2QG, United Kingdom; Department of Orthopaedics, Royal Free Hospital, London NW3 2QG, United Kingdom; Department of Radiology, Royal Free Hospital, London NW3 2QG, United Kingdom

**Keywords:** cystic adventitial disease, meniscus tear, knee

## Abstract

Cystic adventitial disease is a rare cause of calf claudication and is characterized by the development of a mucinous, cystic mass within the outer layer (adventitia) of an artery. The popliteal artery is most commonly affected. Several theories and hypotheses exist regarding the aetiology of this disorder with no clear unifying cause accepted in the literature to date. We describe a case of a 32-year-old female with a one-year history of medial right knee pain and intermittent claudication who was diagnosed with a medial meniscal tear and a large parameniscal cyst communicating with the popliteal artery adventitia. She underwent arthroscopic meniscectomy and cyst decompression and achieved an excellent functional outcome with resolution of the claudication.

## Background

Cystic adventitial disease (CAD) is a very uncommon vascular condition characterized by the accumulation of mucinous or proteinaceous material in the form of a cystic mass within the adventitial layer of a blood vessel.^1^ The incidence is approximately 1/1200 (<0.1%) cases and typically affects middle-aged patients, usually in the absence of atherosclerosis.^2^ The popliteal artery is affected in the majority of cases (85%), leading to symptoms of lower limb claudication and calf pain, and it has very rarely been reported to affect veins in the literature.^3^ The physical examination findings are often normal; however, a popliteal bruit or weakened distal pulses may be exhibited on knee flexion. Diagnosis is frequently supported by imaging in the form of ultrasound doppler, CT angiography, or MRI. The treatment options in the literature have been broadly categorized into percutaneous aspiration of the cyst or vascular surgical options, for example: cyst resection with preservation of the artery or arterial resection with venous graft interposition. We describe an interesting case of a 32-year-old female who was diagnosed with CAD of the popliteal artery secondary to communication with a medial meniscus tear and its associated parameniscal cyst. After a successful arthroscopic meniscectomy and cyst resection under orthopaedic surgery, she achieved an excellent functional outcome. This case thereby supports the synovial theory of cystic adventitial disease with successful outcomes when treating the origin of the cyst.

## Case presentation

A 32-year-old female presented with a one-year history of posterior right knee and calf pain. She described symptoms of intermittent claudication whereby her right calf pain was aggravated by walking long distances and relieved on resting. In addition, she was experiencing medial knee joint pain alongside the claudication. There was no history of trauma. She had no significant medical history or surgical history and was not taking any routine medications.

On clinical examination, she had an active range of motion from 0° to 120° and was tender in the right popliteal fossa with no masses palpable clinically. She had mild posteromedial joint line tenderness. There were no crepitations, and no joint effusion was palpable.

## Investigations

**Figure 1.**
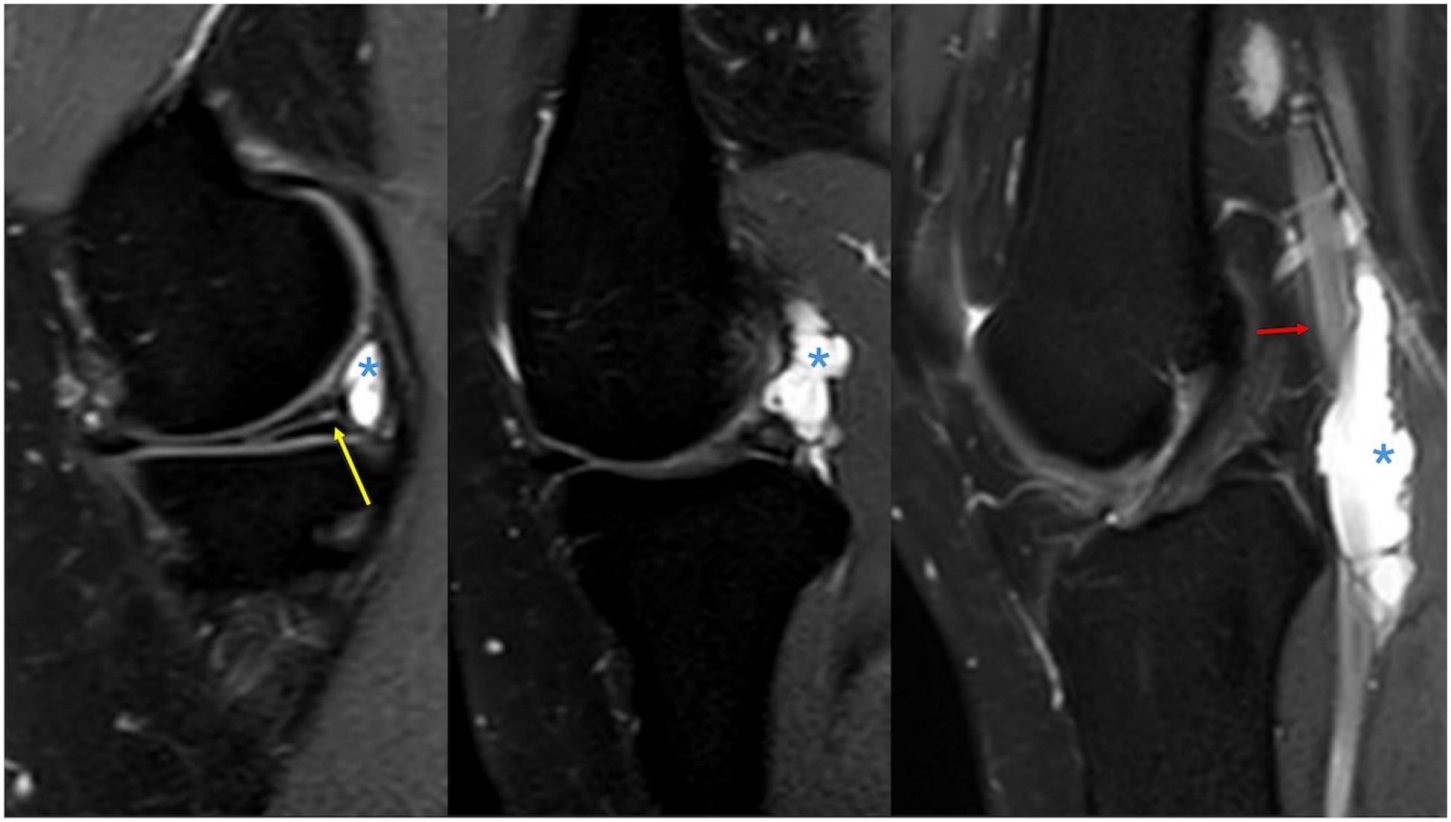
Sagittal proton density fat-saturated sequences demonstrating a horizontal tear involving the body and posterior horn of the medial meniscus (yellow arrow). A large parameniscal cyst appears to arise from this meniscus tear (asterisk) and extends superolaterally into the popliteal fossa and is intimately related to the popliteal artery (red arrow) with longitudinal extension.

**Figure 2.**
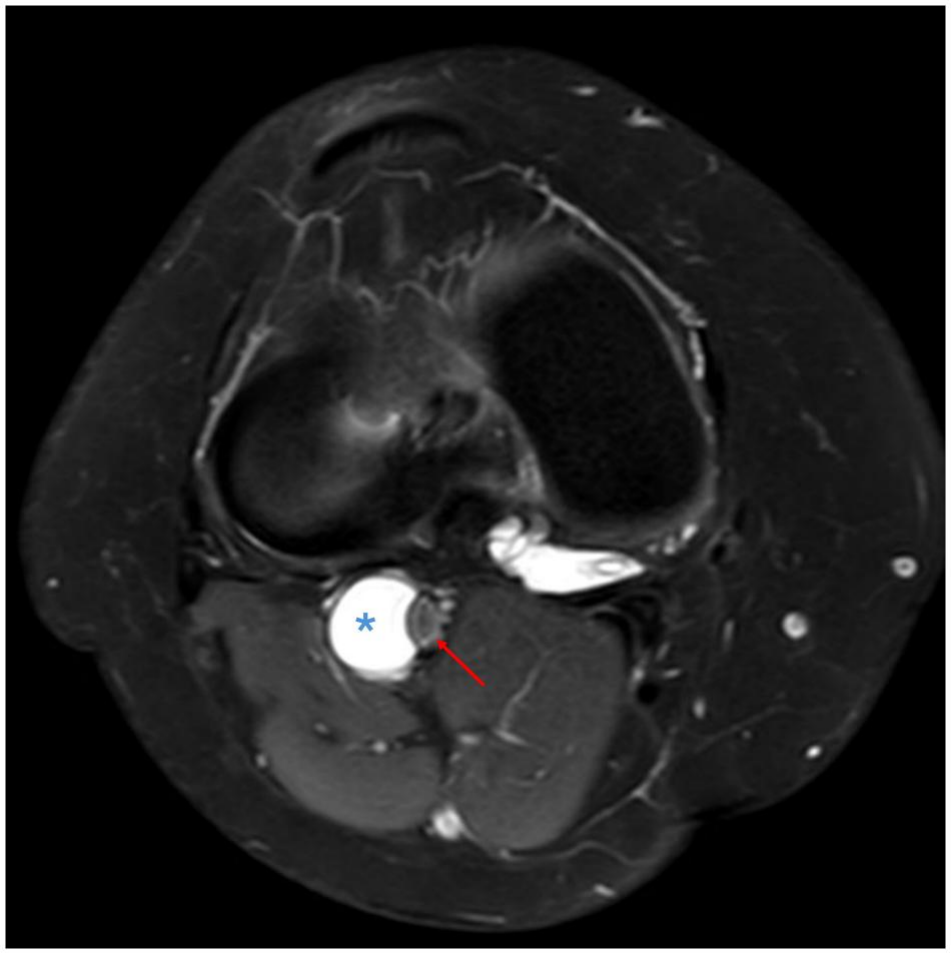
Axial proton density fat saturated sequence illustrating the cyst being related to the outer wall (adventitia) of the popliteal artery (asterisk), measuring approximately 14 mm in diameter, and causing popliteal artery stenosis (red arrow) with preserved flow void.

**Figure 3.**
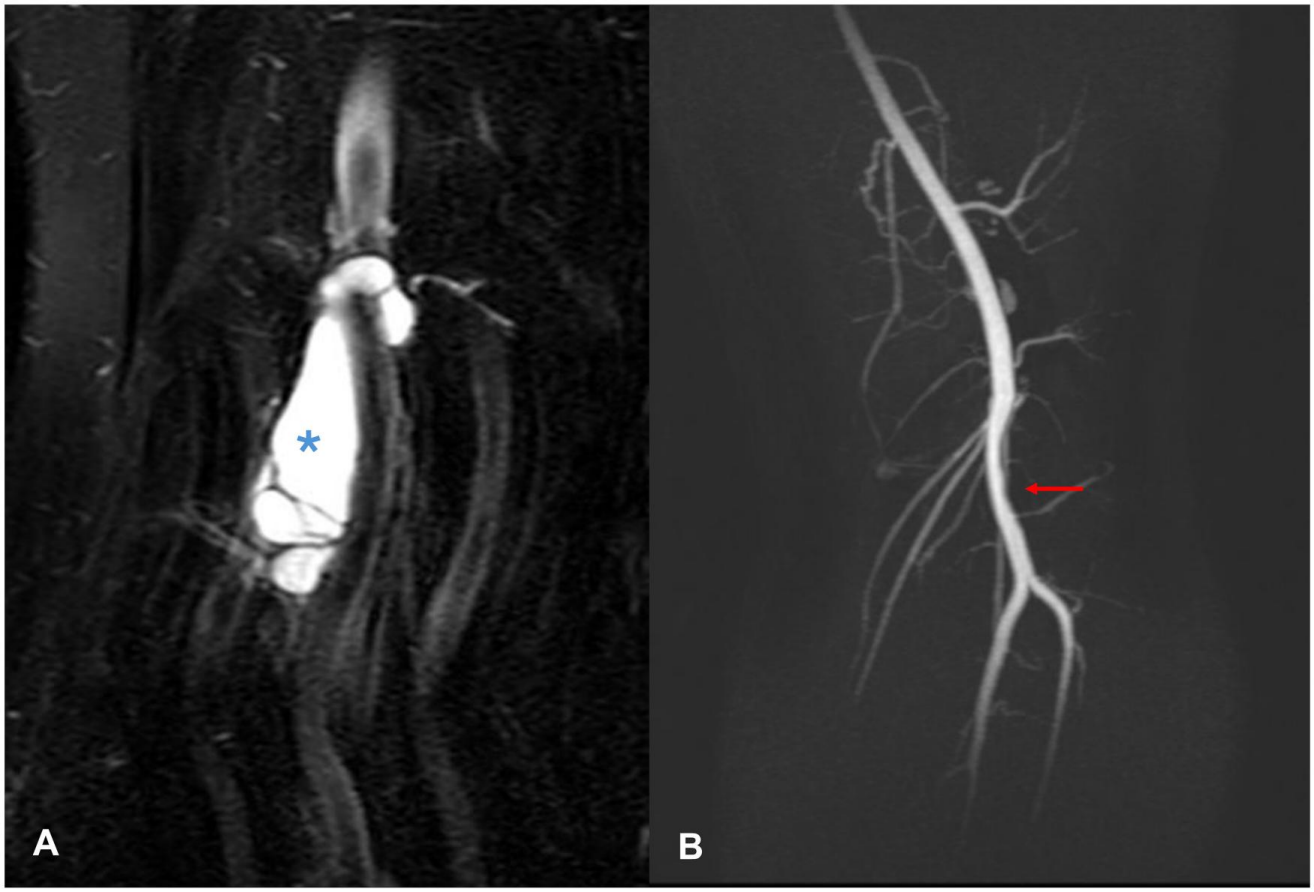
(A) Coronal proton density fat saturated sequence showing an approximately 5 cm coronal length span of the cystic mass within the popliteal artery adventitia (asterisk). (B) Time-of-flight sequence depicting a 5 cm region of stenosis of the popliteal artery (red arrow) correlating with the site of the cyst (scimitar sign).

An MRI knee with time-of-flight (TOF) angiogram sequences demonstrated a horizontal tear involving the body and posterior horn of the medial meniscus within the right knee. A large parameniscal cyst extended from the medial meniscus tear through the posterior capsule supero-laterally into the popliteal fossa. This cyst appeared to communicate and track along the adventitia of the popliteal artery longitudinally over a length of 5 cm, causing subsequent popliteal artery stenosis. MRI TOF angiogram illustrated approximately 50% stenosis of the popliteal artery because of external compression from the parameniscal cyst with no evidence of vessel occlusion (Figures 1-3).

## Treatment

The case was discussed at the local orthopaedic multidisciplinary team meeting in which several management strategies were discussed—for example, percutaneous image-guided aspiration, arthroscopic meniscectomy, and cyst decompression or arteriotomy with cyst resection and grafting under vascular surgery. A decision was made to proceed with partial medial meniscectomy and decompression of the parameniscal/ganglion cyst extending into the adventitia of the popliteal artery. The patient underwent a successful operation and was subsequently referred for physiotherapy and rehabilitation to improve her function.

## Outcome and follow-up

She was reviewed in the orthopaedic clinic 6 months after her operation and described a resolution in her claudication symptoms, significant improvement in her knee pain and improving activities of daily living. A follow-up MRI scan of the right knee 8 months post-surgery demonstrated complete resolution of the popliteal artery CAD and she remains symptom-free 4 years post-surgery.

## Discussion

The first reported case of CAD was in 1947 by Atkins and Key,^4^ and since then, more than 500 cases have been reported in the literature. The popliteal artery is the most commonly affected vessel in the majority of cases; however, there have been cases involving the external iliac, femoral, ulnar, radial, and brachial arteries.^5^ In contrast to peripheral arterial disease, CAD commonly affects middle-aged individuals in the third to fourth decades of life with a 15:1 male to female predilection. The primary symptom experienced is progressive lower extremity claudication. Interestingly, the neurovascular examination findings are often normal; however, reduced distal pulses may be demonstrated on knee flexion due to the local compressive effects of the cyst. In contrast, popliteal entrapment syndrome (a common differential diagnosis) is caused by congenital aberrant anatomy or acquired hypertrophy of the medial head of the gastrocnemius, which contributes to extrinsic compression of the popliteal artery. Therefore, this condition often demonstrates reduced distal pulses on plantarflexion, thereby propagating effects of popliteal artery compression due to calf contraction.

Several theories have been described in the literature to date with no current universally accepted aetiology for CAD. The earliest theory was proposed by Linquette et al.^6^ in 1967, who postulated that de novo mucinous degeneration within the adventitia results from a generalized systemic or connective tissue disorder. The traumatic theory states that repetitive shearing forces and microtrauma to an artery can cause small adventitial detachments, haemorrhage, and cyst formation, thus possibly explaining the development of CAD.^5^ However, recent systematic reviews have questioned this theory, as many cases have no history of trauma.^7^ The developmental theory suggests that during embryogenesis, mucin-secreting mesenchymal cells are incorporated into the adventitia of arteries. Thus, mucin secretion in later life causes progressive cystic enlargement of the adventitia.^8^ Finally, the synovial or articular theory suggests that the aetiology of CAD is due to direct tracking of synovial fluid from joints to nearby vascular structures. This theory is further supported by several cases demonstrating direct intra-articular communication with adventitial cysts on pre-operative imaging, during surgery or both.^7^ In addition, trauma or degeneration to a joint can further propagate migration of synovial fluid due to capsular defects. Our case report supports the synovial theory as pre-operative MRI imaging demonstrated a direct intra-articular communication between the medial meniscal tear, parameniscal cyst, and the adventitial cyst.

Diagnosis of CAD is supported by radiological investigations. The first line test is duplex ultrasonography which demonstrates characteristic findings of a hypoechoic or anechoic cystic mass closely associated with the arterial wall, often causing stenosis to the vessel. Cystic adventitial disease will demonstrate posterior acoustic enhancement (due to liquid contents) and no internal vascular flow on sonography, which can help differentiate this from a popliteal artery aneurysm.^1^ Pre-operative cross-sectional imaging with MRI can assist with surgical planning, as this helps to characterize the morphology of CAD, its relationship with nearby joints and also assists with identifying possible intra-articular communication.^3^^,^^7^ This can be supplemented with non-invasive angiographic techniques using MRI TOF or invasively with digital subtraction angiography to assess the degree of arterial stenosis. Large lesions can cause eccentric stenosis and displacement of the artery due to extrinsic compression contributing to the “scimitar sign.”^8^

The management options for CAD are broadly categorized into conservative, percutaneous aspiration, or surgical intervention. The treatment is ultimately guided by the patient’s clinical presentation, severity of symptoms, and radiological features. Conservative management is considered in patients who do not experience significant discomfort to warrant invasive management as the adventitial cysts may resolve spontaneously in select patients. Percutaneous aspiration is minimally invasive and can be supplemented with image guidance (ultrasound or CT). Although this is associated with fewer complications, there is a high probability of recurrence, and aspiration may be technically unsuccessful in cases where CAD is multiloculated or has viscous contents.^7^ The most common surgical treatment method is cyst evacuation with vein graft bypass/reconstruction, which has generally yielded good results; however, approximately 7% of cases demonstrate persistence or recurrence.^9^ Case reports in the literature have demonstrated early recurrence of CAD in both venous and synthetic grafts.^10^ This evidence suggests that failure to treat the root cause or articular communication of CAD can potentially contribute to re-accumulation of fluid and symptom recurrence due to a persistent joint connection. In our case report, targeted treatment of the intra-articular communication of the cyst (meniscal tear) alongside cyst evacuation yielded a successful outcome with clinical and radiological evidence of complete resolution.

## Learning points

- Cystic adventitial disease is a rare, yet an important differential diagnosis to consider in young- or middle-aged patients with calf claudication in the absence of typical risk factors of cardiovascular disease.
- It may arise due to an intra-articular communication between a meniscal tear of the knee and cystic adventitial disease of the popliteal artery.
- Management options are dependent on symptomatology and radiological features; however, identifying and treating the potential root cause can lead to good functional outcomes.
